# An in-vivo treatment monitoring system for ion-beam radiotherapy based on 28 Timepix3 detectors

**DOI:** 10.1038/s41598-024-66266-9

**Published:** 2024-07-04

**Authors:** Laurent Kelleter, Lukas Marek, Gernot Echner, Pamela Ochoa-Parra, Marcus Winter, Semi Harrabi, Jan Jakubek, Oliver Jäkel, Jürgen Debus, Maria Martisikova

**Affiliations:** 1 Heidelberg Institute for Radiation Oncology (HIRO) and National Center for Radiation Research in Oncology (NCRO), Heidelberg, Germany; 2 Division of Medical Physics in Radiation Oncology, German Cancer Research Centre (DKFZ), Heidelberg, Germany; 3 National Center for Tumor Diseases (NCT), NCT Heidelberg, A Partnership Between DKFZ and University Medical Center Heidelberg, Heidelberg, Germany; 4 ADVACAM s.r.o., Prague, Czech Republic; 5 Department of Physics and Astronomy, Heidelberg University, Heidelberg, Germany; 6 Heidelberg Ion-Beam Therapy Centre (HIT), Department of Radiation Oncology Heidelberg University Hospital, Heidelberg, Germany; 7 Department of Radiation Oncology, Heidelberg University Hospital, Heidelberg, Germany; 8 Clinical Cooperation Unit Radiation Oncology, German Cancer Research Centre (DKFZ), Heidelberg, Germany

**Keywords:** Ion-beam therapy, In-vivo monitoring, Charged nuclear fragments, Timepix3, Applied physics, Radiotherapy, Imaging techniques, Translational research

## Abstract

Ion-beam radiotherapy is an advanced cancer treatment modality offering steep dose gradients and a high biological effectiveness. These gradients make the therapy vulnerable to patient-setup and anatomical changes between treatment fractions, which may go unnoticed. Charged fragments from nuclear interactions of the ion beam with the patient tissue may carry information about the treatment quality. Currently, the fragments escape the patient undetected. Inter-fractional in-vivo treatment monitoring based on these charged nuclear fragments could make ion-beam therapy safer and more efficient. We developed an ion-beam monitoring system based on 28 hybrid silicon pixel detectors (Timepix3) to measure the distribution of fragment origins in three dimensions. The system design choices as well as the ion-beam monitoring performance measurements are presented in this manuscript. A spatial resolution of ${{4}\,\hbox {mm}}$ along the beam axis was achieved for the measurement of individual fragment origins. Beam-range shifts of ${1.5}\,\hbox {mm}$ were identified in a clinically realistic treatment scenario with an anthropomorphic head phantom. The monitoring system is currently being used in a prospective clinical trial at the Heidelberg Ion Beam Therapy Centre for head-and-neck as well as central nervous system cancer patients.

## Introduction

Ion-beam radiotherapy is characterised by its steep dose gradients and high biological effectiveness against various types of cancer^1,2^. While being more expensive than conventional radiotherapy with photons, ion beams are useful for re-irradiation, treatment boosting (dose escalation) and the treatment of radiation-resistant tumours^3^. High precision, however, comes at the cost of proneness to treatment uncertainties^4^. Among others, patient-setup uncertainties and inter-fractional anatomical variations have been identified as important contributors^5^. Usually, patients receive tens of daily treatment fractions for which the patient position is aimed to be reproduced with millimetre accuracy using laser markers and planar X-ray imaging. After the initial treatment-planning CT, control CTs may be used for the identification of potential anatomical variations. However, a control CT may be prescribed if variations are expected by the radiation oncologist. Control CTs only represent a snapshot of the patient anatomy and—if not performed in the treatment room right before irradiation—are unable to resolve patient-setup uncertainties or daily random variations such as, e.g., the sinus cavity filling with mucous^5^. Daily online adaptive ion-beam radiotherapy with advanced 3D imaging techniques such as CT or MRI have so far been too costly and resource-intensive to be implemented in clinical routine^6^.

Secondary radiation from nuclear interactions of the ion beam with the patient tissue has the potential to provide the currently unavailable in-vivo treatment feedback by combining therapy and diagnostics (theranostics). For example, position-emission tomography (PET), prompt-gamma imaging (PGI) and charged nuclear fragments (secondary ions) have been suggested to provide in-vivo treatment verification^7,8^. However, as of 2023, no in-vivo treatment feedback method has so far made the leap from clinical studies into clinical routine in any ion-beam therapy centre worldwide, although PET and PGI systems for proton therapy have come very close recently^9,10^. For carbon-ion beam radiotherapy (CIRT) it has been shown that there are about $20\times$ less prompt gammas emitted compared with proton therapy, effectively ruling out PGI for CIRT unless gamma detection efficiency and signal-to-noise ratio of current detection systems is improved considerably^11^. Concerning CIRT, an offline PET system has been developed at the Heidelberg Ion Therapy Centre (HIT, Heidelberg, Germany) as early as 2013, but has since been decommissioned^12^. An online PET system for carbon-ion beams is being used in a clinical trial at CNAO^13^.

The focus of this work is on in-vivo monitoring of ion beams using charged nuclear fragments. In the case of carbon-ion beams, these secondary ions consist mostly of protons and helium ions^14^. The aim is to measure the fragmentation/interaction vertices (fragment origins) during several treatment fractions and to link observed differences in their distribution to variations in the patient setup or anatomy. A strong advantage of this monitoring technique is that there are virtually no background charged particles. However, a disadvantage is that the fragments undergo Multiple Coulomb Scattering (MCS) of about 1$^{\circ }$ in the patient before reaching the detector and are overwhelmingly emitted in the forward direction, which limits the achievable spatial resolution of the fragment origin measurement along the beam axis^15^. The use of charged nuclear fragments for in-vivo monitoring has been explored in numerous simulation and phantom studies^16–20^. More recently, our group investigated the optimal tracker position and track projection algorithm when using hybrid silicon pixel detectors (Timepix3) as a particle tracker^21,22^.

A full-sized charged-particle tracking detector made of scintillating fibres read out by silicon photo-multipliers (SiPM) was developed within the INSIDE collaboration at CNAO (Pavia, Italy)^23,24^. This system has since been employed in a clinical trial with head-and-neck cancer patients^25^. Here, we report the development of a monitoring system based on 28 Timepix3 detectors, which are organised in seven mini-trackers. The focus of the monitoring system is on patients with cancers of the head-and-neck and the central nervous system (CNS). This manuscript illustrates the design and the performance of this novel in-vivo monitoring system, including the technical (mini-tracker geometry, readout, cooling, mechanical support) as well as analytical aspects (data post-processing, data analysis). Characterisation measurements demonstrate the measurement precision of fragment origins and the detection performance of inter-fractional anatomical changes. The monitoring system is currently being used in the In-Vivo Monitoring (InViMo) clinical trial at HIT.

## Materials and methods

### Heidelberg ion-beam therapy centre

The Heidelberg Ion-Beam Therapy Centre is a synchrotron-based radiotherapy centre that has been treating cancer patients with ion beams since 2009^26^. HIT has two treatment rooms equipped with horizontal beam lines (named H1 and H2), as well as a third room with an ion-beam gantry^27^. The measurements with the developed monitoring system presented here were performed exclusively in H2. Protons as well as helium and carbon ions are currently available for treatment using the active raster-scanning technique with ion pencil beams^28^. The Beam Application and Monitoring System (BAMS) located in each beam nozzle consists of two Multi-Wire Proportional Chambers (MWPC) to measure the pencil-beam position and spot size as well as three ionisation chambers to count the number of delivered ions. This information is stored in Beam Record Files (BRF), which are used in the data post-processing, see Section 2.8.

**Figure 1:**
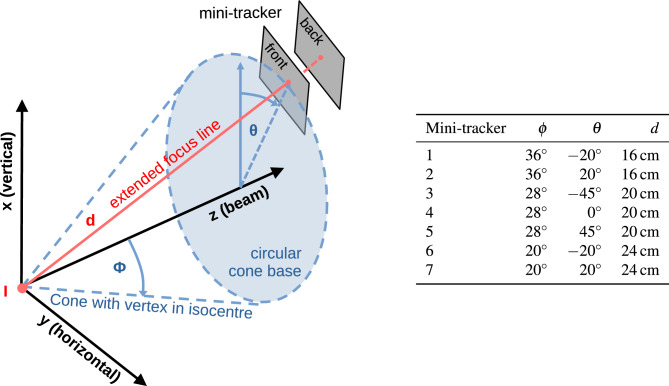
Definition of the mini-tracker positions. The focus line of the mini-tracker is the dotted red line between the central points of the front and back detection layers. The extended focus line runs along the surface of a cone that has its vertex in the isocentre *I*. $\phi$ is the half-angle of the cone, $\theta$ is the azimuth angle of the extended focus line (for $\theta =0$ the focus line is in the x-z plane) and *d* is the distance of the front detection layer to the isocentre.

The coordinate system of the treatment room is centred on the isocentre, which is a defined point along the undeflected path of an ion beam about ${1.4}\,\hbox {m}$ downstream of the vacuum window of the beam line. Its x-axis refers to the vertical axis pointing upwards, the y-axis is horizontal pointing from the left to the right (beam’s eye view) and the z-axis is the beam axis, see Fig. 1.

### Mini-trackers

**Figure 2:**
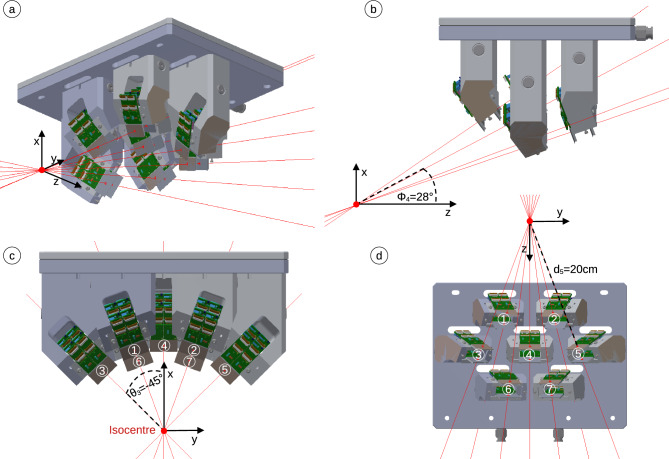
Four different views of the 3D model of the monitoring system with red focus lines and definitions of $\phi _4$, $\theta _3$ and $d_5$ as well as the labels of mini-tracker numbers and the x (vertical), y (horizontal) and z (beam) room axes.

At the heart of the monitoring system are seven mini-trackers (AdvaPIX TPX3 Quad) developed by Advacam s.r.o. (Prague, Czech Republic). A detailed characterisation of the mini-tracker performance concerning the tracking of charged particles has been performed in a previous publication^29^. All specifications of the AdvaPIX TPX3 Quad can be found in the data sheet, which are available from the vendor upon request. A mini-tracker is made of four Timepix3 hybrid silicon pixel detectors, which are arranged in two pairs (two double-sized detection layers) in a telescope setup. Each Timepix3 chip pair connects to a double-sized 300 $\upmu \hbox {m}$-thick silicon sensor with a sensitive surface area of 28 $\hbox {mm}\times \,14\,\hbox {mm}$ equipped with 512 $\hbox {pixel}\,\times \,256\,\hbox {pixel}$ at a pixel pitch of 55 $\upmu \hbox {m}$. That brings the total area of the seven-tracker monitoring system to 7 $\times \,2.8\,\hbox {cm}\,\times \,1.4\,\hbox {cm}$ =27.4$\hbox {cm}^{2}$, see Fig. 2. The mini-trackers use data-driven readout, which makes them effectively dead-time free up to a maximum pixel-signal rate of 47$\hbox {Mhits}\,s^{-1}$. Charged nuclear fragments are detected with almost 100% efficiency and with a time resolution of 1.17 $\hbox {ns}$^29^.

### Monitoring system geometry

In order to harvest the free information about the treatment quality that is carried by the charged nuclear fragments, as many fragments as possible should be tracked. The project budget allowed for seven mini-trackers to be purchased. The aim of the monitoring system design is to find the optimal positions of the seven mini-trackers for in-vivo ion-beam therapy monitoring, taking into account all mechanical, physical, electronic and clinical conditions. Here, we explain the reasoning behind the proposed monitoring system design.

As a clinical prerequisite we focus on patients with tumours in the head that are treated at one of the horizontal beam lines at HIT. Those patients are vulnerable to ion-beam range variations due to the heterogeneous structure of the head with soft tissue, bones and air cavities that can fill or empty with mucous. The monitoring system was hence optimised to fit into the clinical workflow of these particular indications in this treatment room.

A major monitoring system design requirement due to the nature of nuclear interactions is the strongly forward-peaked emission of charged nuclear fragments, which decreases exponentially with increasing observation angle relative to the beam axis^14^. This cone-shaped symmetry of the emission motivates the final arrangement of the mini-trackers. In a previous publication, we explored the influence of the observation angle on the detection significance of an internal density change in a plastic head phantom^21^. It was found that observation angles between 20$^{\circ }$ and 40$^{\circ }$ are the best trade-off between the number of tracked fragments and the spatial resolution along the beam axis of the fragment origins. Moreover, due to the relatively small sensitive area of the employed mini-trackers, they need to be positioned as close as possible to the fragment origins in order to maximise the number of tracked fragments ($d<{30}\,\hbox {cm}$). This condition excludes positioning the mini-trackers conveniently out-of-way below the patient table, as it can be done for prompt-gamma-based in-vivo-monitoring systems^30^. If positioned to the side of the patient, measurements would either be restricted to one patient side or the monitoring system would need to be moved over depending on the patient-table angle. However, a sub-millimetre detector positioning repeatability is required for inter-fractional monitoring because of the acute angles of the mini-trackers relative to the beam axis, which is much easier to realise with a fixed and rigid monitoring system. We therefore decided to position the mini-trackers above the patient. The drawbacks of the measurement position above the patient are twofold: first, the nuclear fragments will almost always cross the oral and nasal cavities on their way to the mini-trackers. Out-of-field variations of the cavity fill status with mucous could therefore produce false-positive signals of inter-fractional density changes, which do not affect the dose distribution in the planning target volume (PTV). We will explore the impact of these variations in a separate manuscript. Second, the support structure of the monitoring system will inevitably limit the patient-table mobility, if the monitoring system is to touch the floor. However, the vast majority of head cancer patients are prescribed at least one treatment field with a patient-table angle near 0 $^{\circ }$ or 180 $^{\circ }$ (i.e. the inferior-superior axis of the patient is perpendicular to the beam). Thus, the monitoring system was designed specifically for these patient-table angles, with a tolerance of approximately ±20$^{\circ }$.

Figure 2 shows the 3D model of the 3D-printed aluminium support structure holding the seven mini-trackers. Figure 1 defines the mini-trackers positions. In order to explain the geometry, we define the focus line of a mini-tracker as the line connecting the centres of the two detection layers. We also define three right circular cones with half-angles of $\phi ={20^{\circ },\,28^{\circ }\,\hbox {and}\,36^{\circ }}$, that have the z-axis (beam axis) as their central axis and their vertex in the isocentre *I*. The seven mini-trackers are arranged in three groups of 2, 3 and 2 modules on the respective cones such that their focus lines run along the cone surfaces and converge at the cone vertices (the room isocentre).

The front-end electronic boards of the mini-trackers point away from the beam axis in order to minimise their suffered radiation damage. The angle $\theta$ denotes the azimuth angle along the circular cone base, with $\theta =0$ referring to the top position when the focus line is in the xz-plane. A maximum value of $\theta ={\pm 45}^{\circ }$ was chosen in order to stay clear of the patient shoulder. Finally, the distance *d* of the front detection layer to the isocentre completes the definition of the mini-tracker positions. The mini-trackers with the largest angle $\phi$ to the beam axis are positioned closest to the isocentre in order to homogenise the number of tracked fragments in each mini-tracker. The total solid angle covered by the mini-trackers is approximately ${0.09}\,\hbox {sr}$.

The table in Fig. 1 lists the positions of all seven mini-trackers. The mini-trackers are numbered from closest to furthest and from left to right (beam’s eye view), with mini-tracker number 4 at the centre, see Fig. 2. Flat cables connect the mini-trackers to their readout interface, which sit above the aluminium support structure. A two-piece 3D-printed plastic cover protects the mini-trackers from external influence.

### Trolley

**Figure 3:**
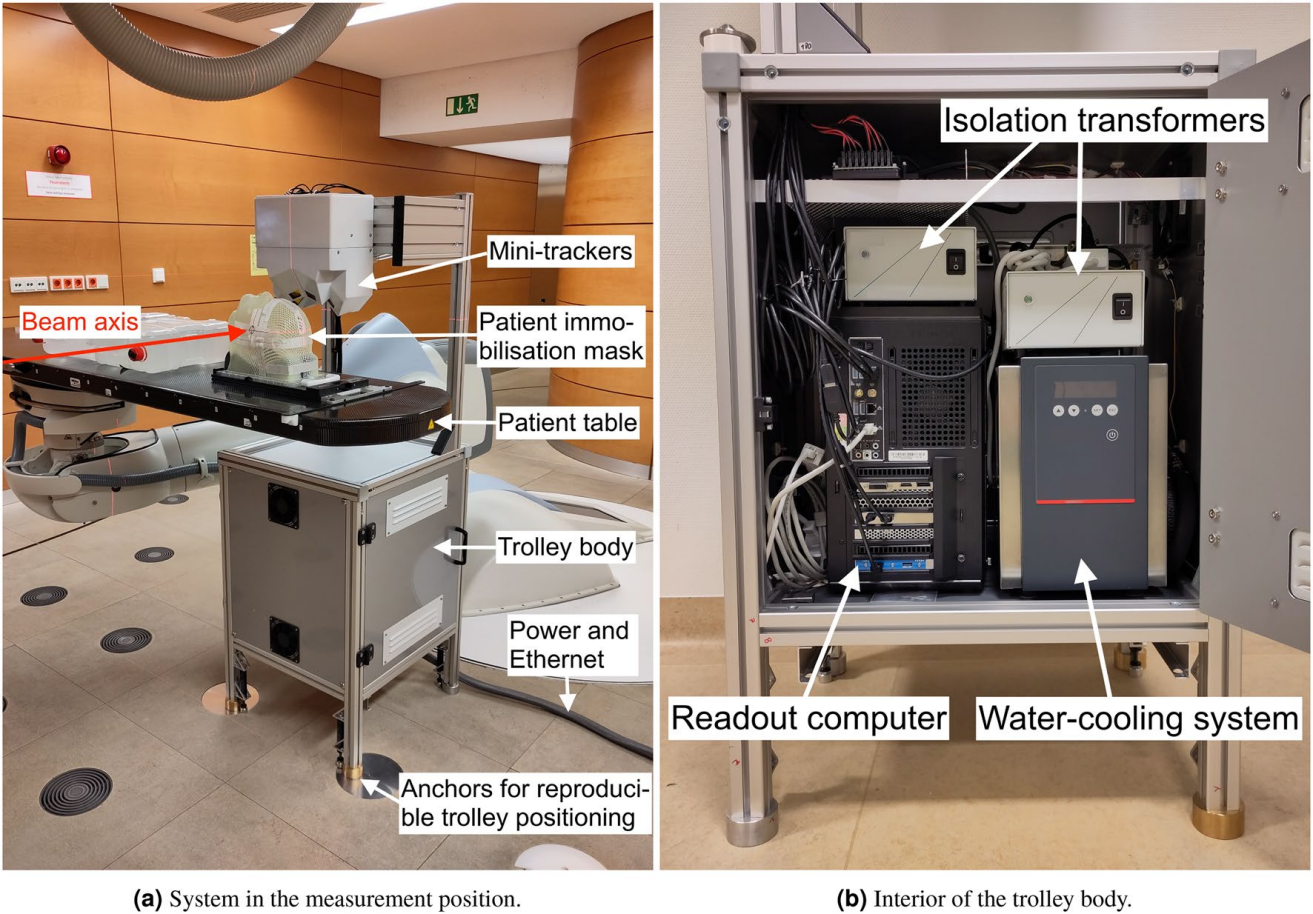
Photographs of the monitoring system with annotations.

The purpose of the trolley is to support the mini-trackers mechanically and to safely house all equipment required for their operation. A photograph of the trolley in the measurement position at HIT is shown in Fig. 3a. In order to achieve maximal robustness and flexibility, the chassis was custom designed of $40\,\hbox {mm}\,\times \,40\,\hbox {mm}$ T-slot aluminium extrusion profiles by the mechanical workshop of DKFZ. The mini-trackers are supported by two vertical aluminium profiles, which extend from the box-shaped trolley body (${580\,\hbox {mm}\,\times \,520\,\hbox {mm}\,\times \,470}\,\hbox {mm}$) below the patient table. Two water pipes, seven power cables and the seven USB3 data transfer cables are guided along these profiles from the trolley body towards the mini-trackers. Two doors on opposite sides provide easy and quick access to the interior of the trolley body, which is shown in Fig. 3b. The trolley is supported by four legs, two of which connect via removable pins to aluminium anchors that reach through the loose floor tiles and are screwed into the solid concrete floor below. A combination of round and slit-shaped pins ensures that the trolley slides into a reproducible position without jamming. The trolley is moved in and out of the measurement position with a lifting table. A single power cable and an Ethernet cable for remote control of the readout PC are the only external connections to the monitoring system.

### Data readout

The AdvaPIX TPX3 readout interfaces of the individual mini-trackers as well as their synchronisation were developed by Advacam. Data transfer is done via USB3 with a maximum readout speed of ${0.32}\hbox {GB}\,\hbox {s}^{-1}$. For the simultaneous readout of seven mini-trackers we opted for a custom high-performance computer (Alpharis Technology GmbH, Freiburg, Germany) with 11 high-speed USB3 ports (read and write speed from ${0.47}\,\hbox {GB}\,\hbox {s}^{-1}$ to ${1.93}\hbox {GB}\,\hbox {s}^{-1}$). This PC was optimised for the parallel readout of several Timepix3 detectors at full load, which requires a main board with sufficient PCIe links between the USB3 ports and the CPU. Moreover, the PC is equipped with ${256}\,\hbox {GB}$ RAM and a ${16}\,\hbox {TB}$ SSD for fast data handling (read and write speed of ${5}\,\hbox {GB}\,\hbox {s}^{-1}$ and $12\,\hbox {GB}\,\hbox {s}^{-1}$, respectively). An AMD Threadripper 3960X processor with 24 cores and a Nvidia 1050 graphics card (${4}\,\hbox {GB}$ RAM) allow fast data post-processing and analysis.

### Safety

The monitoring system was designed with a focus on patient and staff safety. To meet the safety standards (including IEC 60601 and §126 StrlSchV), a risk assessment was carried out with the involvement of all concerned professions (medical physicists, medical-technical radiology assistants and radio-oncologists) before the start of clinical activities. The resulting risk mitigation measures were implemented in accordance with HIT’s internal quality management system.

No component of the monitoring interferes with the primary ion beam, neither physically nor electromagnetically. There are no moving parts in the entire setup. The extension of the beam path downstream of the isocentre is left free of any material in order to avoid unnecessary induced radioactivity in the monitoring system.

To ensure electrical safety of the patient and the personnel handling the monitoring system, two isolation transformers are used. Similarly, a network isolator protects against leakage currents. All electric devices are connected to the earth provided by the isolation transformers via the conductive trolley chassis.

Moreover, influence tests were performed in order to make sure that the presence of the monitoring system does not affect the readings of the BAMS through electromagnetic fields or mechanical vibrations. The results showed no relevant interaction between the monitoring system and the BAMS.

A measurement workflow was developed with the aim to minimise the interference with the clinical treatment workflow at HIT and guarantee the safety of the patient and the medical and technical staff. As a consequence of the optimisation, the additional time that the patient spends in the treatment room was reduced to less than five minutes per treatment fraction.

### Temperature management

Temperature management is an important issue for the stable operation of the Timepix3 detectors^31^. Most importantly, individual pixels may become noisy with increasing temperature, which could occupy a significant part of the readout bandwidth. The heat production of a single mini-tracker at the maximum data rate is ${8}\,\hbox {W}$, i.e. ${56}\,\hbox {W}$ for the entire monitoring system. Therefore, the 3D-printed aluminium support structure of the mini-trackers was designed to be water-cooled from the inside. Water cooling is provided by an air-cooled thermoelectric Huber Piccolo chiller (Peter Huber Kältemaschinenbau SE, Offenburg, Germany) with a cooling power of ${280}\,\hbox {W}$ at 20$^{\circ }\hbox {C}$. The readout PC uses its own water-cooling circuit for temperature management. The inside of the trolley is air-conditioned by four ventilators (two blowing air in, two out) and four passive ventilation grilles.

### Data post-processing

The Timepix3 raw data comes in the form of text files with each line representing a pixel signal. Listed are the pixel signal coordinates, time of arrival (ToA, ${40}\,\hbox {MHZ}$ clock frequency), fast time of arrival (FToA, ${640}\,\hbox {MHZ}$) and time over threshold (ToT, ${40}\,\hbox {MHZ}$ clock frequency)^32^. Custom-written data post-processing routines (Matlab 2021, MathWorks, Natick, Massachusetts, USA) are used to process pixel signals to reconstructed fragment tracks in 3D^21,22^. First, the pixel timestamps are built from ToA and FToA and the pixel ToT is calibrated to energy deposition using pixel-wise calibration matrices provided by the vendor^32,33^. Next, collections of neighbouring pixel signals (including diagonals) with a maximum time difference between consecutive pixel signals of ${500}\,\hbox {ns}$ form clusters. The cluster energy deposition is defined as the sum of the individual pixel energy depositions and the cluster position is the energy-deposition-weighted mean of the pixel positions. The earliest pixel signal timestamp is defined as the cluster timestamp. Coincident clusters in the front and back detection layers of a mini-tracker with a maximum timestamp difference of ${\pm 75}\,\hbox {ns}$ (to account for potential ToA clock miscounts) form a reconstructed fragment track.

A pencil-beam line is characterised by its spot position along x and y and by its timestamp, as measured by the BAMS in the beam nozzle. The different timelines of the BAMS pencil-beam data and the Timepix3 fragment data are synchronised offline during data post-processing so that each fragment track can be assigned to a pencil-beam line based on their coincident timestamps. The pencil-beam line and the fragment track form two skew lines that do generally not cross. We approximate the fragment origin (also called fragmentation vertex or interaction vertex) as the mid-point along the shortest connection between the pencil-beam line and the fragment track. This method is called the closest-distance projection algorithm and was previously found to yield the best results for the measurement of the longitudinal (along the z-axis or beam axis) fragment origin coordinate^22^.

### Fragment-origin distribution analysis

In order to perform inter-fractional in-vivo monitoring, the fragment-origin distributions of two measurements (treatment fractions) are compared in 3D. The coronal and axial planes were selected for data visualisation because they show best the signal introduced by the silicone flap on the cranial half of the PTV. Two different analysis metrics are used in this work: the absolute difference in the number of measured fragments in the coronal plane and the two-sample Kolmogorov-Smirnov (KS) statistical test in the axial plane^34^. The KS test quantifies the likelihood that the two measured samples originate from the same probability distribution: for that, it defines the maximum difference between the normalised cumulative histograms as a test statistic and converts the result to a p-value. Because of the normalisation, the KS test is sensitive to changes of the histogram shape but not to the absolute number of detected fragment tracks. More advanced analysis methods to optimise the sensitivity of the monitoring system to inter-fractional density changes are currently being investigated.

### Characterisation measurements

**Figure 4:**
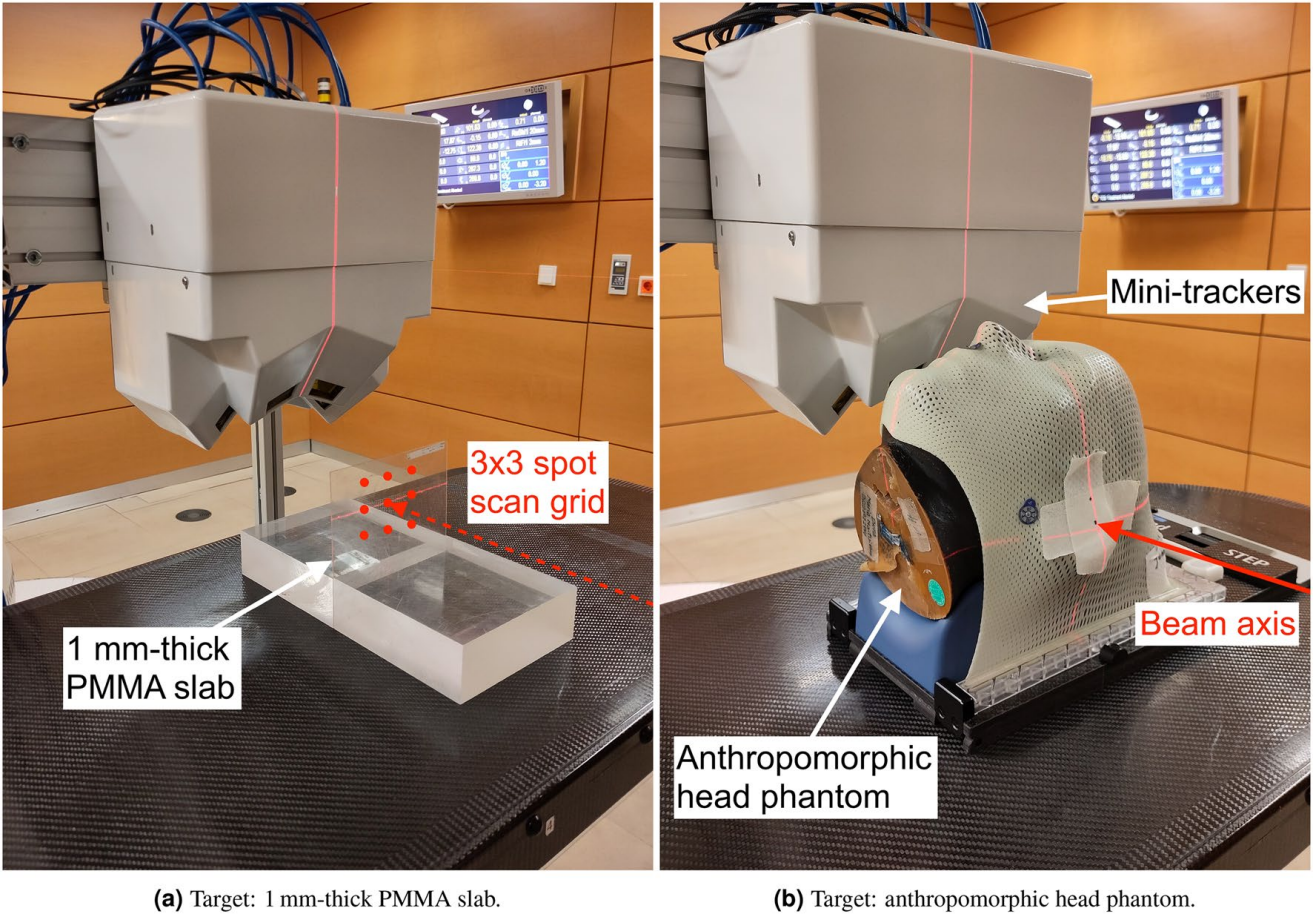
Photographs of the characterisation measurements of the monitoring system. Nine pencil beams on a $3\times 3$ scan grid were used to irradiate the thin PMMA slab. A treatment plan for a spherical target volume was designed for the anthropomorphic head phantom.

Two different characterisation measurements were performed as part of this work. First, a ${1}\,\hbox {mm}$-thick PMMA slab at the isocentre was irradiated in order to demonstrate the precision of the fragment-origin measurement in 3D, see Fig. 4a. Nine carbon-ion pencil beams on a $3\times 3$ spot scan grid centred on the isocentre were irradiated. The distance between neighbouring spots on the grid is ${20}\,\hbox {mm}$ and the spot size is ${3.4}\,\hbox {mm}$ FWHM. Two million carbon ions were irradiated per spot, with a kinetic energy of ${430.1}\,\hbox {MeV}\,\hbox {u}^{-1}$. This corresponds to a peak physical dose in PMMA of ${1.5}\,\hbox {Gy}$ at the centre of each pencil beam, according to the treatment planning software Raystation version 11B (RaySearch Laboratories AB, Stockholm, Sweden).

**Figure 5:**
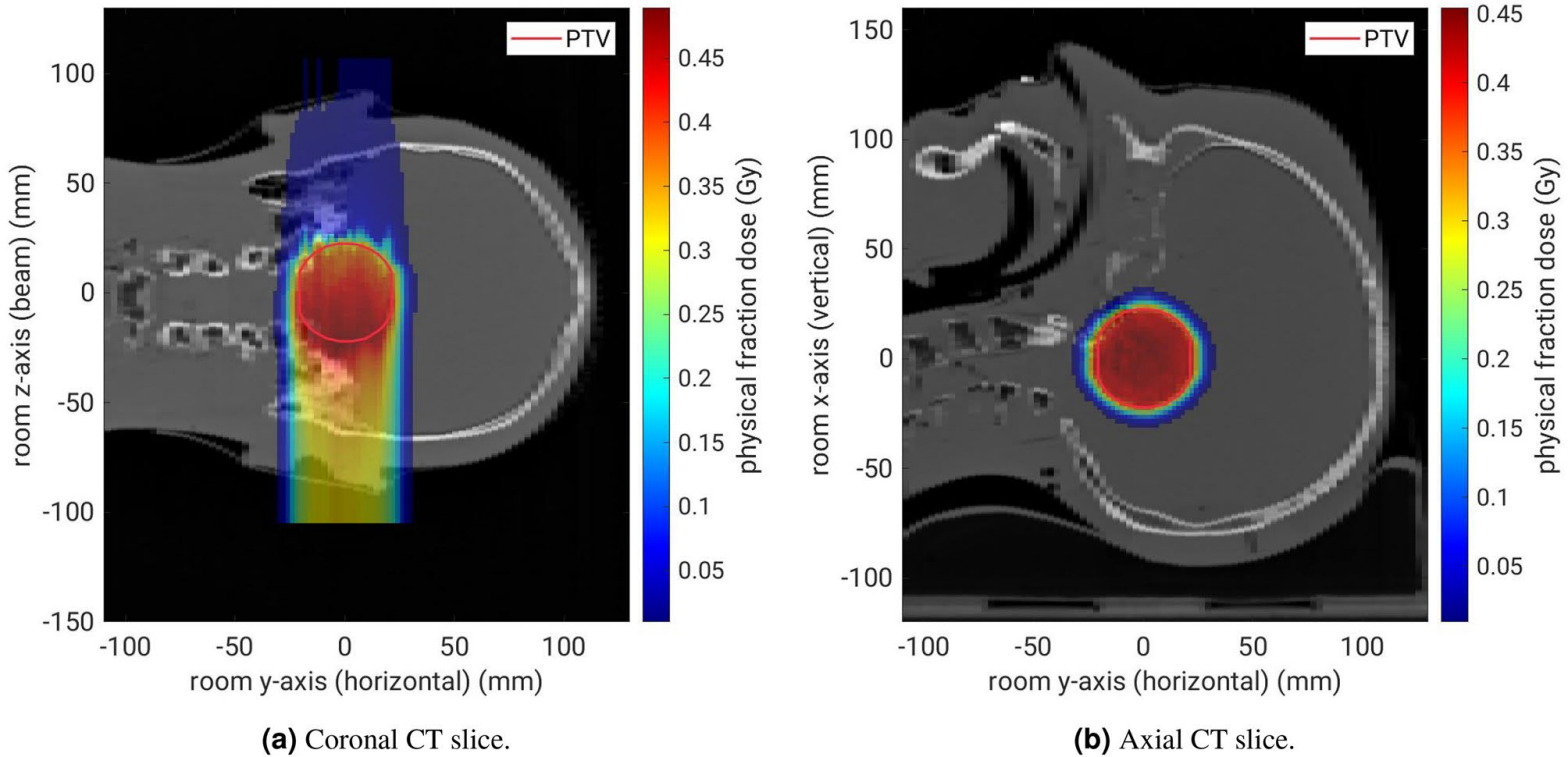
Isocentric CT slices with the planned single-fraction physical dose distribution in the anthropomorphic head phantom. The red circle delineates the spherical PTV near the base of skull.

As a second characterisation measurement, we performed a carbon-ion treatment fraction of a virtual spherical target volume of ${50}\,\hbox {mL}$ volume at the centre of an anthropomorphic Alderson head phantom (Radiology Support Devices, Inc., Long Beach, California, USA). This measurement aims to reproduce a realistic clinical patient case with a base-of-skull tumour as close as possible. A photograph of this measurement setup is shown in Fig. 4b. The CT scan of the head phantom was performed on a SOMATOM Sensation Open CT scanner (Siemens Healthineers AG, Erlangen, Germany) at the Heidelberg University Hospital following the standard protocol for patients. The treatment plan was calculated using the Syngo RT Planning software (Siemens Healthineers AG). The plan consists of two opposing treatment fields (patient-table angles of 0 $^{\circ }$ and 180$^{\circ }$) with a combined biologically effective dose of 3 Gy(RBE). Only the field with a patient-table angle of 0$^{\circ }$ was irradiated in this work. The planned physical dose distribution of this field is shown in Fig. 5. The field contains $2.16\,\times \,10^{8}$ primary carbon ions with ion energies from 166 to ${255}\,\hbox {MeV}\,\hbox {u}^{-1}$. Two irradiations were performed where the first measurement acts as a reference for the expected fragment yield. In the second irradiation, a ${1.5}\,\hbox {mm}$-thick water-equivalent silicone flap (Troll Factory Rainer Habekost e.K., Riede, Germany) was added to cover the cranial half of the target area, resembling a pull-back of the beam range via tissue swelling at the skin. Such swellings are commonly seen in radiotherapy patients, for example because of a weight change over the course of the month-long treatment, a reaction to radiation or from a build-up of fluid due cortisol administration. The aim of this second characterisation measurement is to demonstrate the system’s performance at detecting relative changes in the patient anatomy based on measured fragment origins in a realistic clinical setting.

**Figure 6:**
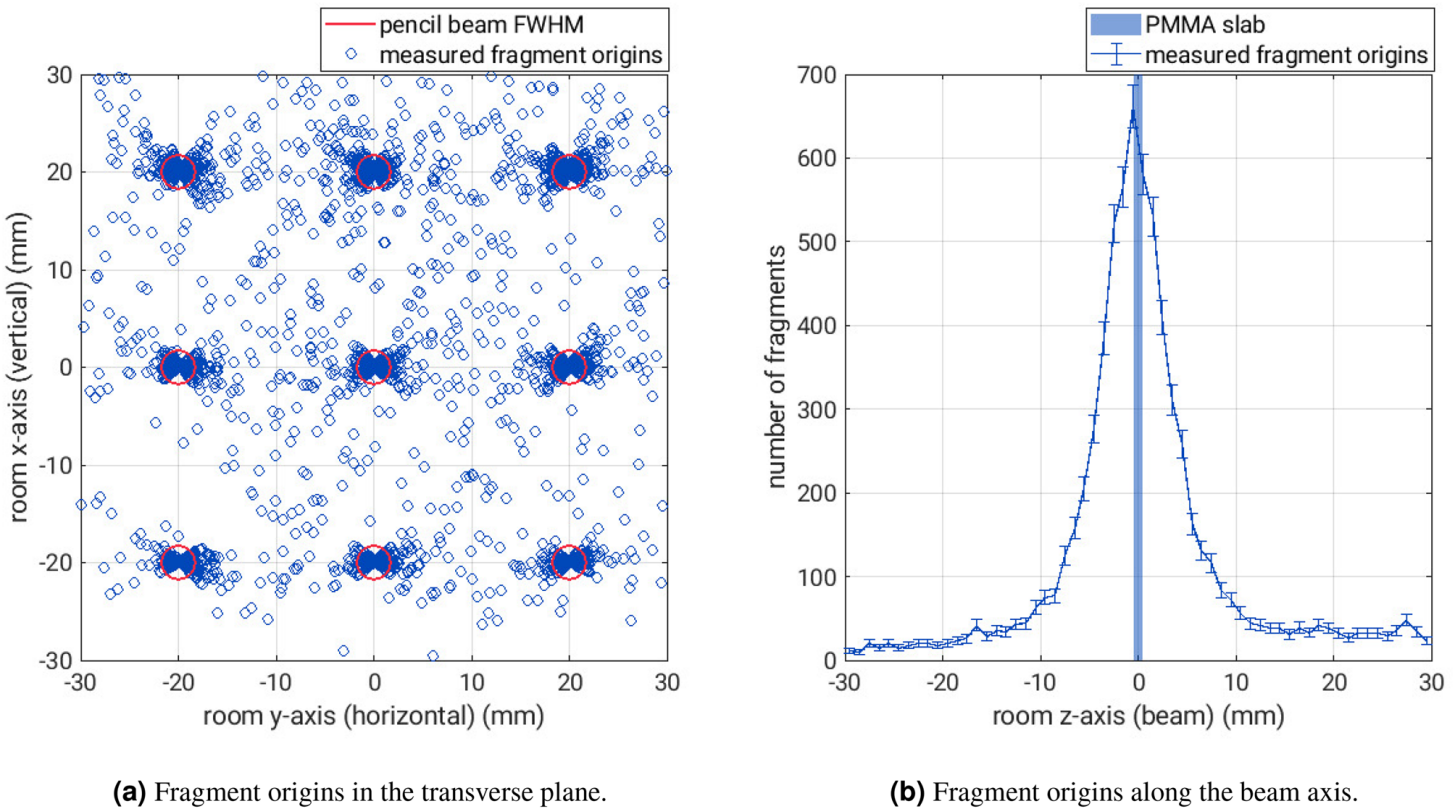
Fragment-origin measurement from the ${1}\,\hbox {mm}$-thick PMMA slab in three room dimensions. The red circles in (**a**) represent the FWHM spot size of the pencil beams. The blue-coloured area in (**b**) represents the thickness of the PMMA slab.

## Results

### Precision of the fragment origin measurement

Figure 6 shows the measured fragment origins from the ${1}\,\hbox {mm}$-thick PMMA slab in three dimensions. In total, approximately 8000 fragment tracks were detected from the PMMA slab. Figure 6a shows a scatter plot of the measured fragment origins in the transverse xy-plane. The red circles represent the pencil-beam spot size in air of ${3.4}\,\hbox {mm}$ FWHM at the nominal pencil-beam positions. More than 85% of the measured fragment origins fall within this nominal pencil-beam spot size, while the FWHM is defined as containing about 58% of a two-dimensional Gaussian distribution. This observed bias is inherent to the utilised closest-distance projection algorithm because it uses the nominal pencil-beam position as an input parameter for the fragment origin reconstruction. As a consequence, the measured fragment origins of an individual mini-tracker follows the shape of an arc that is centred on the nominal pencil-beam positions. Combining the data from seven mini-trackers results in the butterfly shape observed in Fig. 6a. An alternative projection method to get a more accurate fragment distribution in the xy-plane is to use the prior information of the fragment emission depth by calculating the intersection point of the fragment track with a plane at a fixed depth (here: $z=0$).

Figure 6b shows a histogram of the z-coordinates of the measured fragment origins. Here, the measured spatial resolution is $\sigma ={4}\,\hbox {mm}$, even though the PMMA slab thickness is only ${1}\,\hbox {mm}$. The spatial resolution is dominated by the pencil-beam spot size and amplified by the acute detection angle relative to the beam axis (mini-tracker angles are from 20$^{\circ }$ to 36$^{\circ }$). If the spatial resolution along the z-axis is evaluated for every mini-tracker individually, the two mini-trackers at $\phi ={36}^{\circ }$ achieve an average of $\sigma ={3}\,\hbox {mm}$, while the mini-trackers at $\phi ={20}^{\circ }$ have an average spatial resolution of $\sigma ={5}\,\hbox {mm}$. All mini-trackers reach their sharpest spatial resolution for the bottom row of pencil beam spots ($x={-20}\,\hbox {mm}$) because these fragment tracks form the largest angle to the beam axis (angle $\phi$ in Fig. 1).

### Anthropomorphic head phantom

**Figure 7:**
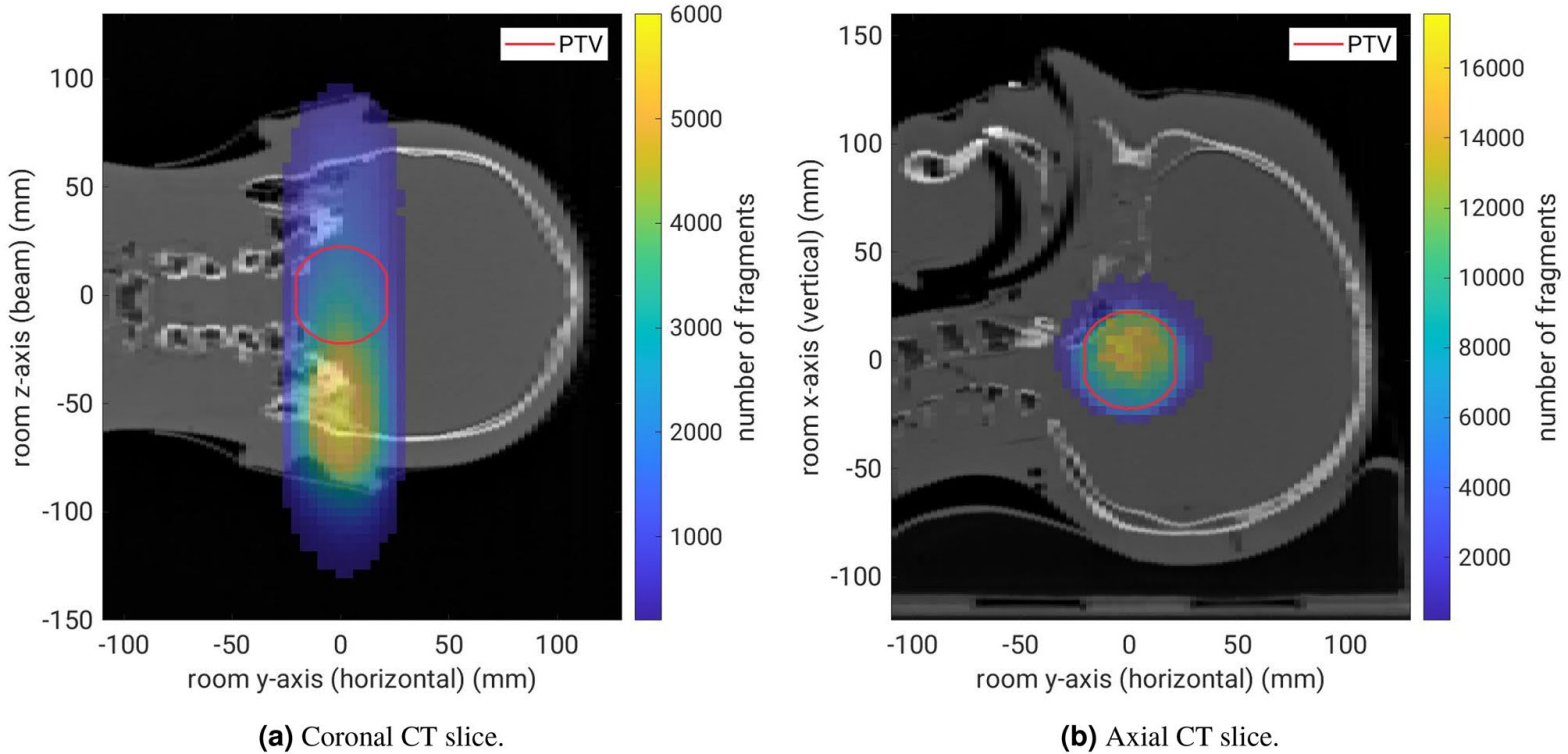
Fragment-origin distribution measured by the monitoring system in the isocentric coronal and axial planes. The measured fragment origins are integrated along the axis perpendicular to the shown plane.

Figure 7 shows the 2D histograms of the measured fragment origins in the isocentric coronal and axial planes. The data are integrated along the third axis, thus showing all measured fragment origins in both plots. In total, $1.4\,\times \,10^{6}$ fragment tracks were detected from the head phantom. Most detected fragments originate from the beam-entrance side of the head phantom, as expected from previous measurements^17^. The limited average spatial resolution along the z-axis of ${4}\,\hbox {mm}$ for individual fragments leads to some fragments seemingly emerging from the air upstream of the head phantom. Figure 7b shows also the detection bias for fragments originating from the anterior patient side where the monitoring system is situated. This is explained by the decreased absorption of these fragments on their way to the detection system, as well as their more acute detection angle.

**Figure 8:**
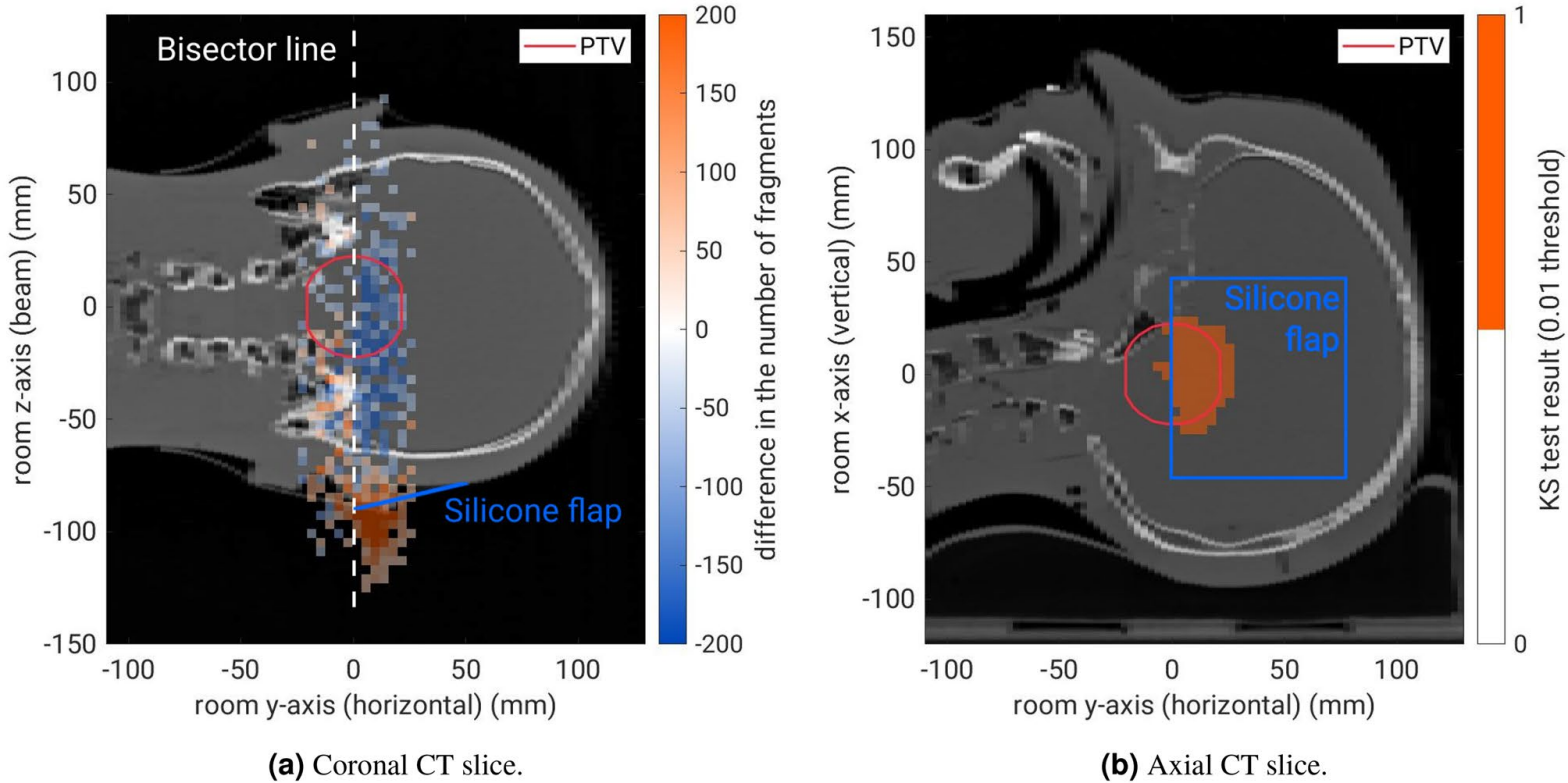
(**a**) Coronal view of the difference in the number of measured fragment origins between the reference measurement and the measurement with a ${1.5}\,\hbox {mm}$-thick silicone flap covering the cranial half of the PTV (red circle). (**b**) Axial view of the KS statistical test result (p-value threshold of 0.01) between the reference and the silicone flap measurement.

Despite the limited spatial resolution along the beam axis, the silicone flap produces a significant signal in the measured fragment-origin distributions. This is shown in Fig. 8, where a measurement with and without a ${1.5}\,\hbox {mm}$-thick silicone flap covering the cranial side of the treatment plan area are compared. Figure 8a shows the difference between the two integrated fragment origin histograms in the coronal plane. A bisector line divides the distributions into two equal halves with (cranial side) and without (caudal side) the presence of the silicone flap. The influence of the silicone flap is clearly visible on the cranial side of the bisector line as a relative shift of the two longitudinal histograms, which manifests as an over-production of fragments upstream of the production peak and an under-production downstream of it. From the observed longitudinal distribution shift, the thickness of the silicone flap can be calculated: using a $\chi ^2$ minimisation, it was measured to be ${1.54}\,\hbox {mm}$ (expectation value of ${1.50}\,\hbox {mm}$). The left side without silicone flap only shows difference variations within the statistical uncertainty with a measured longitudinal distribution shift of ${0.04}\,\hbox {mm}$ (expectation value of ${0}\,\hbox {mm}$).

Figure 8b shows the result of the KS statistical test (p-value threshold of 0.01) in the axial plane. Again, the presence of the silicone flap is evident with the KS test yielding a positive result (KS test result of 1) on the cranial side of the bisector line and a negative result (KS test result of 0) on its caudal side. In the course of this study it was found that the KS test yields more significant results compared with a $\chi ^2$ test for the detection of anatomical changes. These results prove the sensitivity to small shifts of the fragment origins along the beam axis.

## Discussion

### Monitoring system design

The presented device is the first clinically-used semiconductor-based monitoring system dedicated for in-vivo monitoring of ion-beam radiotherapy with charged nuclear fragments. A major advantage is its modular design: in theory, the number of mini-trackers is only limited by the available space around the patient. The current cooling system theoretically allows for up to 35 mini-trackers to be cooled to 20 $^{\circ }\hbox {C}$. In the current system, the strictest imminent limitation on the number of mini-trackers is the number of full-speed USB3 ports in the readout computer (currently 11).

The flexibility of the module arrangement also allows the adaption of the monitoring system to different tumour sites. The presented system was optimised for the measurement of tumours in the head as well as for treatment fields that are almost perpendicular (${\pm 20}^{\circ }$ tolerance) to the median plane of the patient. If, in the future, the focus is put on other indications, the mini-trackers can be moved altogether or their individual arrangement could be changed. The latter would require a redesign of the module support structure. An update of the trolley would be required in the case that more oblique patient table angles are of interest, because the supporting vertical aluminium elements currently limit the patient-table mobility. Introducing additional flexibility in the system, however, would come at the expense of reduced positioning reproducibility, which is crucial for inter-fractional treatment monitoring. The current monitoring system relies on the rigidity of the trolley and the immutability of the mini-tracker positions to guarantee positioning repeatability.

### Precision of the fragment-origin measurement

While the accuracy of the fragment-origin measurement is determined by the absolute positioning accuracy of the mini-trackers, its precision (the spatial resolution) is dominated by the angular resolution of the fragment track measurement. The angular resolution translates into the spatial resolution of the fragment-origin measurement depending on the angle of the fragment track to the beam axis. The angular resolution is dominated by the the mini-tracker geometry (the precision of the cluster position measurement and the distance between detection layers) and the Multiple Coulomb Scattering (MCS) of the fragments in the patient. The angular resolution of a mini-tracker with ${0.055}\,\hbox {mm}$ cluster position measurement precision (conservatively approximated as the pixel pitch) and ${20.3}\,\hbox {mm}$ distance between the detection layers is below ${0.1}\,^{\circ }$. Meanwhile, the characteristic MCS angle of a ${200}\,\hbox {MeV}$ proton in ${10}\,\hbox {cm}$ of water is approximately 1 $^{\circ }$, which is therefore the dominant contribution to angular resolution^15^.

The choice of the projection algorithm also plays a role for the spatial resolution as different algorithms produce different kind of artefacts, which were explored in a previous publication^22^. For an unbiased xy-coordinate measurement (plane perpendicular to the beam axis), the fragment tracks could be projected onto a fixed $z=const.$ plane. While this is possible for the characterisation measurement with a thin PMMA slab at a known depth, it cannot be done in a thick phantom where the true z-coordinate of a fragment origin is unknown. Anyway, the focus of this work on ion-beam range monitoring is on the measurement of the z-coordinate (beam axis) of the fragment origins, for which the utilised closest-distance projection algorithm was found to be ideal^22^. However, this algorithm ignores the pencil-beam spot size by approximating the transverse origin of every fragment to be the pencil-beam line.

The spatial resolution along the z-axis of the monitoring system was measured to be ${3}\,\hbox {mm}\,to\,{5}\,\hbox {mm}$, which is a result of the amplification of the pencil-beam spot size and the MCS by the acute detection angles of the fragment tracks. This is a design choice of the monitoring system: we sacrificed longitudinal spatial resolution for an increased amount of data because this proved to be more effective for the detection inter-fractional density changes^21^.

### Detection of inter-fractional anatomical changes

The selected carbon-ion treatment scenario is clinically realistic and representative for a tumour near the base of skull treated at HIT. The result shows that the detection and quantification of a superficial ${1.5}\,\hbox {mm}$ range shift is feasible with the monitoring system. A ${1.5}\,\hbox {mm}$ dose shift is half of the setup safety margin in the head used at HIT of ${3}\,\hbox {mm}$. Therefore, the sensitivity of the monitoring system is deemed sufficient to detect clinically relevant dose shifts in ion-beam radiotherapy. The reported results are in line with publications of competing research groups, who report similar detection significance for a similar superficial density change (filling of the sinus cavity)^25^. However, the detection significance of any inter-fractional density change depends on its size and location: the smaller (meaning both thickness and transverse area) and the deeper the change, the harder it is to resolve^21^. A more systematic analysis of the signature of internal density changes of different sizes and positions is in preparation. The influence of density changes located outside of the primary beam but in the path of the fragment tracks towards the monitoring system—which do therefore not change the dose distribution in the PTV but the signal in the monitoring system—is also part of future work. The monitoring system is currently being used in a clinical study with CNS and head-and-neck cancer patients at HIT. If successful, the monitoring system could be used as a trigger for an additional control CT, when an internal density change of relevant magnitude is detected.

## Conclusion

A novel system for in-vivo carbon-ion treatment monitoring was designed, constructed and characterised. It is the first monitoring system to use several hybrid silicon pixel detector modules (Timepix3), which makes it relatively compact and easily scalable. The presented system was optimised for tumours in the head but could also be used for different indications with some geometric adaptions. Custom solutions were developed for the mini-tracker arrangement, temperature management as well as data readout, processing and analysis. A bespoke measurement workflow minimises the interference with the clinical treatment such that the additional time that the patient spends in the treatment room is less than five minutes per treatment fraction. Characterisation measurements with a thin PMMA slab show a spatial resolution of ${4}\,\hbox {mm}$ along the beam axis. A performance test with a clinic-like treatment plan for an anthropomorphic head phantom demonstrates that the monitoring system is sensitive to shifts of the fragment origins along the beam axis of at least ${1.5}\,\hbox {mm}$. The monitoring system is currently being used in the InViMo clinical trial at the Heidelberg Ion Beam Therapy Centre.

## Data Availability

The data will be made available upon reasonable request to Maria Martisikova: m.martisikova@dkfz-heidelberg.de.
